# Gene expression profiling in equine polysaccharide storage myopathy revealed inflammation, glycogenesis inhibition, hypoxia and mitochondrial dysfunctions

**DOI:** 10.1186/1746-6148-5-29

**Published:** 2009-08-07

**Authors:** Eric Barrey, Elodie Mucher, Nicolas Jeansoule, Thibaut Larcher, Lydie Guigand, Bérénice Herszberg, Stéphane Chaffaux, Gérard Guérin, Xavier Mata, Philippe Benech, Marielle Canale, Olivier Alibert, Péguy Maltere, Xavier Gidrol

**Affiliations:** 1 Unité de Biologie Intégrative des Adaptations à l'Exercice -INSERM 902, Genopole Evry, France; 2 INRA, UMR 703, Ecole Nationale Vétérinaire de Nantes, France; 3 INRA, UMR1313 Génétique Animale et Biologie Intégrative, F-78350 Jouy-en-Josas; 4 GenoSciencePharma, 2, rue Mascaron, 13006 Marseille, France; 5 CEA, Laboratoire d'Exploration Fonctionnelle des Génomes (LEFG), Genopole Evry, France

## Abstract

**Background:**

Several cases of myopathies have been observed in the horse Norman Cob breed. Muscle histology examinations revealed that some families suffer from a polysaccharide storage myopathy (PSSM). It is assumed that a gene expression signature related to PSSM should be observed at the transcriptional level because the glycogen storage disease could also be linked to other dysfunctions in gene regulation. Thus, the functional genomic approach could be conducted in order to provide new knowledge about the metabolic disorders related to PSSM. We propose exploring the PSSM muscle fiber metabolic disorders by measuring gene expression in relationship with the histological phenotype.

**Results:**

Genotypying analysis of GYS1 mutation revealed 2 homozygous (AA) and 5 heterozygous (GA) PSSM horses. In the PSSM muscles, histological data revealed PAS positive amylase resistant abnormal polysaccharides, inflammation, necrosis, and lipomatosis and active regeneration of fibers. Ultrastructural evaluation revealed a decrease of mitochondrial number and structural disorders. Extensive accumulation of an abnormal polysaccharide displaced and partially replaced mitochondria and myofibrils. The severity of the disease was higher in the two homozygous PSSM horses.

Gene expression analysis revealed 129 genes significantly modulated (p < 0.05). The following genes were up-regulated over 2 fold: IL18, CTSS, LUM, CD44, FN1, GST01. The most down-regulated genes were the following: mitochondrial tRNA, SLC2A2, PRKCα, VEGFα. Data mining analysis showed that protein synthesis, apoptosis, cellular movement, growth and proliferation were the main cellular functions significantly associated with the modulated genes (p < 0.05). Several up-regulated genes, especially IL18, revealed a severe muscular inflammation in PSSM muscles. The up-regulation of glycogen synthase kinase-3 (GSK3β) under its active form could be responsible for glycogen synthase (GYS1) inhibition and hypoxia-inducible factor (HIF1α) destabilization.

**Conclusion:**

The main disorders observed in PSSM muscles could be related to mitochondrial dysfunctions, glycogenesis inhibition and the chronic hypoxia of the PSSM muscles.

## Background

Several cases of metabolic myopathies have been observed in the Norman Cob horse breed used to pull carriages [1]. Muscle histology examinations revealed that one bloodline suffers from a glycogenosis described as polysaccharide storage myopathy (PSSM) in Quarter horses [2,3] and classified as non-exertional myopathies with rhadomyolysis in equine muscle disorders [4]. The disease has also been observed in Andalusian horses (Spanish purebred horses) [5], Belgian Draft Horses, Morgan, Arabian, Standardbred, ponies, Warm-blooded horses [6] and a mule [7]. The prevalence of PSSM among overtly healthy Quarter Horses in the United States is likely to be between 6% and 12% [8]. A PSSM phenotype has been characterized in a Norman Cob horse pedigree and in a population of stallions, by histological demonstration in striated muscular fibers of an accumulation of some periodic acid Schiff (PAS)-positive amylase-resistant polysaccharides appearing ultrastructurally as glycogen-like particles [1,9]. Using this criteria, the affection prevalence was 33% among a sample of French stallions. The prevalence of PSSM in Norman Cob stallions was closer to the 36% PSSM affected horses diagnosed in Belgian Draft Horses [10] than the 8% observed in draft horses in the United Kingdom [11]. The common clinical signs of PSSM in various breeds are abnormal hind limb gait, poor muscling, generalized muscle atrophy, poor performance, back soreness, exercise intolerance, spontaneous decumbency with inability to rise, episodic "colic" and rhabdomyolysis [12].

In Humans, 11 types of glycogenosis (or glycogen storage diseases) have been described, each containing several sub-types [13]. The genes responsible for glycogen storage diseases in humans [13] or involved in the glycogen pathway were identified in the PubMed data basis and could be considered as candidates for equine PSSM: G6PC, SLC37A4, NPT4, GAA, AGL, GBE1, GBE2, PYGM, PYGL, PFKM, PHKA2, PHKB, PHKG2, PHKA1, PHKG1, GYS1, GYS2. However, in equine PSSM, the aetiology and physiopathology are not known. The excessive glycogen storage and formation of abnormal polysaccharide in PSSM horses therefore appears to reflect an increase in glycogen synthesis rather than a decrease in its utilisation [3]. Glucose tolerance tests showed that PSSM horses have enhanced cellular uptake of glucose and an increased sensitivity to insulin [14]. Neither glycogenolytic or glycolytic enzyme deficiencies, nor abnormality in the phosphofructokinase (PFKM) regulation, have been identified in affected horses [2,15]. The enhanced insulin sensitivity in PSSM horses is not due to an increase of the glucose transporter GLUT4 content or insulin receptor quantity [16]. Attempts to measure branching enzyme activities with methods developed for human muscle were unsuccessful [2]. Linkage studies did not demonstrate the relationships between PSSM and the candidate genes AMPK [17,18] or AGL [19]. A specific form of fatal glycogen storage disease was observed in a Quarter horse foal where a nonsense mutation in codon 34 of the glycogen branching enzyme (GBE1) was identified [20]. This metabolic myopathy can be compared to human glycogenosis type IV where several mutations and deletions explain the total or partial deficiency of the GBE1 gene. In adult horses, a new G-to-A mutation within the gene encoding glycogen synthase 1 (GYS1) has recently been observed in Quarter horses and some warm-blooded horses [21]. It is assumed that the metabolic disorders of PSSM muscles could be observed in detail at the transcription level because the gene(s) responsible for glycogen storage disease would also be linked to other dysfunctions and to some failure in gene regulatory networks. Thus, the functional genomics approach could be conducted in order to provide new knowledge about the metabolic disorders related to PSSM. For a better understanding of the PSSM physiopathology in Norman Cob horses, we propose exploring the PSSM muscle fiber metabolic disorders by measuring gene expression in relationship with the histological phenotype.

## Methods

### Horses, genotyping and muscle biopsies

All the horses were genotyped for the GYS1 c.926G>A mutation [21] (wild type GG, heterozygous GA or homozygous AA).

A biopsy of the *gluteus medius *muscle was performed in 13 Norman adult horses: 4 females and 9 males, 4–17 years old. This muscle was chosen because of (i) its important propulsion function in horse locomotion, (ii) its early and major histological involvement in the course of the disease [1] and (iii) the good standardization of biopsy sampling [22]. The horses were sedated and received a local anaesthesia before making a small skin incision according to a standardized protocol used in routine for muscle disease diagnostic. The biopsy of the dorsal compartment of the *gluteus medius *was performed at the first third distance between the sacral and *coxae tuber*. A biopsy needle with automatic sampling was vertically inserted until its extremity just under the fascia corresponding to a 3.5 cm depth for the *gluteus medius*. Each muscle biopsy was divided in two parts, and the first part was frozen in isopentane cooled with liquid nitrogen for histological analysis and the second part was collected for genomic analysis. The blood was removed by absorbing it with a compress and immediately frozen in the RNA later^® ^at -80°C. Informed consent was obtained from the horse owner, the French National Studs (Les Haras Nationaux), and the study was approved by the animal care committee of the National Research Institute of Agriculture (INRA).

### Histological analysis

Briefly, the muscle samples were serially sectioned (8 mm thick) and each section was stained with either hematoxylin-eosin-safranine (HES), or periodic acid-Schiff (PAS) with or without incubation for 20 minutes at 37°C with α-amylase [1]. Intracytoplasmic presence of amylase-resistant material was used as diagnosis criteria for PSSM: seven were PSSM horses and six were control horses [2]. Morphometric analysis was performed additionally for histologic quantitative evaluation using a digital camera (Nikon DXM 1200, Badhoevedo, Netherlands) combined with image-analyzing software (Lucia imaging software, Laboratory Imaging Inc., Prague, Czech Republic). Percentages of aggregate-containing and vacuolated fibers were calculated as described previously [1] from each PAS-amylase-stained and HES-stained sections respectively.

Additional biopsies from one PSSM horse (#49) was designated for routine electron microscopy and subsequently fixed in 2.5% buffered glutaraldehyde, postfixed in osmium tetroxide, embedded in epoxy resin, cut into 1-mm-thick sections, stained with uranyl acetate and lead citrate, and examined by transmission electron microscopy.

### Production of the equine long oligonucleotide microarray

Gene expression analysis was performed using a homemade equine long oligonucleotide microarray which included 384 equine transcripts: 50 probes of the mitochondrial genome and 334 probes of the nuclear genome [see Additional file 1 and GEO Platform accession number GPL8349: ]. This equine oligonucleotide microarray was designed according to the derived method used for open access human and mouse long oligonucleotide microarrays [23]. Briefly, the list of human annotated genes (available in the NCBI public data base) involved in muscle structure, contraction, energetic metabolism and inflammation processes was used to identify the equine candidate genes that could be interesting to put on the microarray. Then, this list of candidates was crossed with the orthologous equine sequences available in 2006 on the NCBI data base using a blast method [24]. In addition, 50 other probes of the equine mitochondrial genome were designed to measure the expression of the 13 genes (2 different probes per protein gene) coding for the protein units of the respiratory chain as well as 22 tRNA and 2 rRNA. Finally, 384 probes of 50 nucleotides long were calculated and synthesized with a 5' terminal – NH2 modification in order to attach the probe on the slides. The microarrays were spotted at the microarray service (LEFG, CEA, Evry, France) by printing the equine probes, suspended in a spotting buffer composed of 50% dimethyl sulfoxide (DMSO) and TE on hydrogel slides (Schott Nexterion), with a Microgrid-II-robot. The equine microarray can be spotted alone on a slide or jointly with the 25 K mouse oligonucleotide microarray [23].

This equine microarray has been validated by a reproducibility test, competition test, biological test for equine transcript analysis extracted from different muscles of different equine breeds and health status [unpublished data]. Fourteen microarrays were used for analysis of each PSSM muscle sample against reference control muscles using two fluorochromes in a dye-swap design (duplicate measurements).

### RNA extraction

Total RNA was extracted from tissues by a phenol-chloroform method (TRIZOL^®^ reagent). The quality of total RNA was verified by micro channel electrophoresis (RNA 6000 Nano LabChip^®^, Bioanalyzer^®^). The total RNA concentration and RNA purity were measured by optical density with a spectrophotometer at 260 nm and the ratio 260/280 (Nanodrop^®^). RNA was considered as pure when the 260 nm out of 280 nm absorbance ratio was close to 2. The RNA were stored at -80°C.

### RNA in vitro transcription

The synthesis of RNA with a polyA tail was performed using in vitro transcription with aminoallyl UTP. A total RNA (1 μg) was used to start the synthesis using the in vitro transcription kit according to the manufacturer's protocol (Aminoallyl MessageAmp^® ^II aRNA). This method equally increased the number of mRNA copies of each transcript without changing the proportion of the initial population of transcripts. The integrity and concentration of aminoallyl RNA (aaRNA) were checked again by micro channel electrophoresis (RNA 6000 Nano LabChip^®^, Bioanalyzer^®^) and a spectrophotometer (Nonodrop^®^). The aaRNA were stored at -80°C.

### Microrray hybridization and signal quantification

All the samples of PSSM muscles were hybridized against the reference control muscles. This reference was made by pooling together all the mRNA extracted after in vitro transcription from the 6 control muscles of the sound horses. Briefly, the hybridization protocol [24] included the following steps. After purification of the amino-modified RNA, monofunctional forms of the Cyanine 3 (Cy3) or Cyanine 5 (Cy5) fluorochrome were coupled to 5 μg of the studied sample and control sample by an indirect method: the ester groups linked to the fluorochromes were bound on the amino-allyl groups incorporated on the RNA during the in vitro transcription. The marked targets were then mixed and hybridized on the same chip for immediate comparison of the two nucleic acid populations. For each sample, a dye-swap (duplicate measurement) was performed to avoid asymmetry of marker affinity (Cy3 and Cy5). The two results were averaged before statistical results.

The 5 μg of aaRNA were completely vacuum-dried and resuspended in 9 μL of coupling buffer and 11 μL of dye, the tubes were mixed by vortexing and incubated for 30 minutes in the dark. The reaction was quenched by addition of 0.73 M Hydroxylamine and incubation for 15 minutes in the dark. Excess dye was removed from the labeled aaRNA by purification on a column. Purified labeled aaRNA were subsequently mixed with 10 μg poly (A) RNA, 10 μg yeast tRNA, 10 μg Cot 1 mouse RNA, then precipitated with 0.5 volumes of 7.5 M ammonium acetate and 2.5 volumes of absolute ethanol. After centrifugation at 16,000 × g for 45 minutes to 1 hour at 4°C, the supernatant was removed and the pellet was washed once with 700 μL of 70% ethanol. The samples were centrifuged at 16000 g for 15 minutes at 4°C. The aaRNA pellet was vacuum-dried for 1 minute and then dissolved in 50 μL of the hybridization mix preheated at 50°C: 2 × SSC, 0.1% SDS and 0.1% Salmon sperm DNA. It was mixed by vortexing and incubated in a water bath at 50°C to dissolve the mRNA pellet. Each slide was rehydrated in a humidified chamber for 1 hour at room temperature then dried in a dessicator for 1 hour. The slides were prehybridized in 0.3% Ethanolamine and 0.05 M Na Borate pH9. The slides were plunged 1 minute in water and dried by centrifugation for 7 minutes at 100 × g. The probe was heated for 2 minutes at 100°C and placed between the slide and a cover slip. The arrays were incubated overnight in a water bath at 50°C in a humidified slide chamber and washed in a first solution of 0.1 × SSC with 0.1% SDS for 5 minutes followed by two washing steps in 0.1× SSC for 5 minutes each at room temperature, under agitation and safe from the light. The arrays were dried by centrifugation at 100 × g for 7 minutes. The image of fluorescence intensities of Cy3 (532 nm) and Cy5 (635 nm) were scanned separately with the laser scanner (GenePix^® ^4000B).

The tiff images (10 μm/pixel) were analyzed using a specific software for microarray image analysis (GenePix^® ^Pro 6.0 software) which allowed locating the spots automatically with manual control and quantifying the fluorescence intensity of each spot. For each microarray, the data output were the fluorescence intensities of all the spots corresponding to each gene. These data were imported in a genomic data analysis software (Gene Spring^® ^software) to normalize the data before filtering and statistical analysis. For each sample (horse), a duplicate analysis (dye-swap) was made and the average fluorescence results were used. Then, all the results were normalized per spot and per microarray by the Lowess regression method in order to remove systematic variations. Finally, for each sample and each gene an expression ratio was obtained:

Ratio = Expression of each PSSM muscle/expression in the reference control muscle

For statistical analysis, a log transformation ratio was used to normalize the distribution. A list of the significant genes were obtained by filtering the genes with a cut-off t-test p-value p < 0.05 with Benjamini and Hochberg multiple testing correction method to avoid a false discovery rate [25]. The Student t-test detected the mean expression ratio of replicates which are significantly different from one (expression ratio = 1, means no gene expression change).

### Real time quantitative RT-PCR

In order to validate the equine microarray results, quantitative RT-PCR were conducted in triplicate for the top ten up and down-regulated gene transcripts both in control and PSSM horses. The primers were designed using Primer express software (Applied Biosystem) (Table 1). All the reactions were carried out on the real time PCR system 7500 (Applied Biosystems). Briefly, reverse transcription was performed on 20 ng of aaRNA obtained by aminoallyl amplification (RNA used for microarray hybidization and RT-PCR were issued from the same preparation). Random primers (100 ng) and 10 pmol of dATP, dCTP, dGTP and dTTP were added to RNA in a 13 μL final volume. The reaction tubes were incubated at 70°C for 5 min. The random annealing primer was performed by an incubation at room temperature (~25°C) for 10 min. Four microliters of 5× first strand buffer, 200 pmol of DTT and 200 U of Superscript II reverse transcriptase were added in the tube for a 20 μL final volume and incubated at 42°C for 1 hour. The enzyme was inactivated by incubating the mixture at 70°C for 15 min. The cDNA concentration was measured by optical density with a spectrometer that measured absorbance at 260 and 280 nm (Nanodrop^®^). The equine cDNA solution was diluted in water to obtain a final concentration of 20 ng/μL and stored at -20°C. Complementary DNA (100 ng) obtained from aaRNA was amplified in a 20 μL PCR reaction with 10 μmol of each specific primer and 10 μL SYBR^® ^Green PCR Master Mix. Amplification steps were as follows: 10 min denaturation at 95°C, 40 cycles of denaturation at 95°C for 15 s and annealing extension at 60°C for 1 min. The comparative threshold cycle (CT) method was used for the calculation of amplification as specified by the manufacturer. The expression results of the quantitative RT-PCR of the control and PSSM horses were normalized by the mean CT of the reference gene 12S rRNA measured in all the muscle samples and the relative expression ratio of PSSM against the control group was calculated.

**Table 1:** List of the primers used for real time quantitative RT-PCR.

**Gene**	**Primer name**	**Primer sequence**	**Length**	**Salt Tm**	**GC%**
IL18	IL18_1Fwd	TGCATTAGCTTGGTGGAAATG	21	61	42
	
	IL18_1Rev	AGGTTCAAGCCTGCCAAAGT	20	61	50

CTSS	CTSS_1Fwd	ACAACGGCATCGATTCAGAC	20	61	50
	
	CTSS_1Rev	GCCAAAGGGAAGTTCGGTAT	20	60	50

LUM	LUM_1Fwd	CCTGGAGGTCAATGAACTTGAA	22	61	45
	
	LUM_1Rev	ACGCAAATGCTTGATCTTGG	20	61	45

CD44	CD44_1Fwd	GATCCACCCCAACTCCATCT	20	61	55
	
	CD44_1Rev	TGACCGAGGTGCAGTCTTCT	20	61	55

FN1	FN1_1Fwd	CCCTGGTGTCACAGAGGCTA	20	61	60
	
	FN1_1Rev	AGTTGGGGAGGCTCATCTGT	20	61	55

GSTO1	GSTO1_1Fwd	TACCTCATCTGGCCCTGGTT	20	62	55
	
	GSTO1_1Rev	ATGGCTTCCATCCAGAGCTT	20	61	55

VCAM1	VCAM1_1Fwd	GCACTGCCATTGAGATGTGA	20	61	50
	
	VCAM1_1Rev	TGCAATGTTGCTATGGCTCA	20	61	45

ITGB1	ITGB1_1Fwd	TCTCCAGAAGGTGGCTTTGA	20	61	50
	
	ITGB1_1Rev	TCCGTGGAAAATACCAGCAG	20	61	50

CXCR4	CXCR4_1Fwd	GCAGCAGCAGGTAGCAAAGT	20	61	55
	
	CXCR4_1Rev	CCACGTCATCCTCCGTGTAG	20	61	60

HIF1A	HIF1A_1Fwd	GGACTCGGATCATCTGACCA	20	61	55
	
	HIF1A_1Rev	TATCCCCCTCTTTTGGCAAG	20	61	50

NFKBIB	NFKBIB_1Fwd	CGTCATCCACAAAGATGCAG	20	60	50
	
	NFKBIB_1Rev	CTGGGCCTCAACAGCCTAGT	20	62	60

PLCG2	PLCG2_1Fwd	CATCTTGTACGGCACCCAGT	20	61	55
	
	PLCG2_1Rev	TTCAAGCCAGACAGCCACTT	20	61	50

ND3	ND3fwd	CCACAACTAAACATCTATGCAGAAAAA	27	62	33
	
	ND3rev	GGAGGCGTGCTGACCCTAT	19	62	63

SLC25a17	SLC25a17_1Fwd	GCAATCTGGGTCAAAGGTCA	20	61	50
	
	SLC25a17_1Rev	GCCCCTTGCAGCTTTAGTCT	20	61	55

SRI	SRI_1Fwd	ATTGCCGGAGGATACAAACC	20	61	50
	
	SRI_1Rev	GCCATTCAGTACAGCCCAGA	20	61	55

ND5	ND5fwd	GATGATGATACGGCCGAACAG	21	61	52
	
	ND5rev	CCCGATGCGGTTATAAAGGA	20	58	50

PDE4D	PDE4D_1Fwd	AGCCTGCGAACTGTACGAAA	20	61	50
	
	PDE4D_1Rev	ATGGATGGTTGGTTGCACAT	20	61	45

SLC25a15	SLC25a15_1Fwd	TGAACTGAGCCGATCGTTTT	20	61	45
	
	SLC25a15_1Rev	CAGAGGCAGATTCCACCAAC	20	61	55

VEGF	VEGF_1Fwd	TCTACCTCCACCATGCCAAG	20	61	55
	
	VEGF_1Rev	GTCTCGATTGGACGGCAGTA	20	61	55

PRKCA	PRKCA_1Fwd	GACTTCATGGGCTCCCTTTC	20	61	55
	
	PRKCA_1Rev	TCTTCGTCCCCTTCTGGAAT	20	61	50

SLC2A2	SLC2A2_1Fwd	GCCATCGGTACTCTTCACCA	20	61	55
	
	SLC2A2_1Rev	TGTGCCACAGCTTCTGATTG	20	61	50

### Data mining

A biological interpretation of the data was performed using two types of analysis to identify the main biological functions and the main metabolic pathway disorders revealed by the expression profile of the PSSM muscles. The categorization of the gene functions was generated through the use of Ingenuity^® ^Pathway Analysis . The analysis identified the biological functions that were the most significant to the gene list results. The significant genes associated with biological functions in the knowledge database (gene functions described in published papers) were considered for the analysis. The Fischer exact test was used to calculate a p-value determining the probability that each biological function assigned to that data set is due to chance alone.

The analysis of the main metabolic pathways and their dysfunctions was performed using a Java/Perl software, Predictsearch^® ^(Prediguard, ) which has been previously described in [26]. This software allows the characterization of the pathways as well as the functional networks in which the selected genes were involved. Briefly, gene aliases corresponding to the significant modulated genes were submitted as queries to PubMed in order to collect titles and abstracts of all related publications. The results are presented in a 2D graph that shows the relationships between each gene and cell type, cellular compartment, and biological function.

## Results

### Genotypes and histological phenotypes

The PSSM horse genotypes for the GYS1 c.926G>A mutation were 5 heterozygous (AG) and 2 homozygous (AA) (Table 2). The 6 control horses were wild type (GG). Heterozygosity seemed to be consistent with the dominant mode of inheritance.

**Table 2:** Genotype of GYS1 mutation and histological data of the horses.

**Horse**	**PSSM disease status**	**Genotype**	**Histological grading**	**Age (years)**	**Sex**	**Vacuolized fibers (%)**	**Inflammation**	**Necrosis**	**Central nuclei**	**Anisocytosis**	**Lipomatosis**	**Aggregate containing fibers (%)**
10	0	GG		16	M	ND						0

29	0	GG		15	M	0						0

35	0	GG		8	M	0						0

40	0	GG		8	M	0						0

56	0	GG		6	F	0						0

34	0	GG		10	M	0						0

38	PSSM	GA	1	12	M	14.1	mild	mild	numerous	mild	none	2.5

54	PSSM	GA	1	4	F	2.5	none	none	several	minimal	mild	2.4

12	PSSM	GA	2	17	M	7.3	mild	mild	numerous	mild	mild	20.6

32	PSSM	GA	2	6	M	1.4	mild	none	several	mild	none	17.0

42	PSSM	GA	2	9	F	ND	minimal	mild	several	mild	mild	9.9

44	PSSM	AA	2	17	F	21.9	mild	severe	several	severe	mild	10.5

49	PSSM	AA	2	10	M	34.1	severe	severe	numerous	severe	severe	18.9

Genotypes: GG = wild type, GA = heterozygous, AA = homozygous

**Figure 1:**
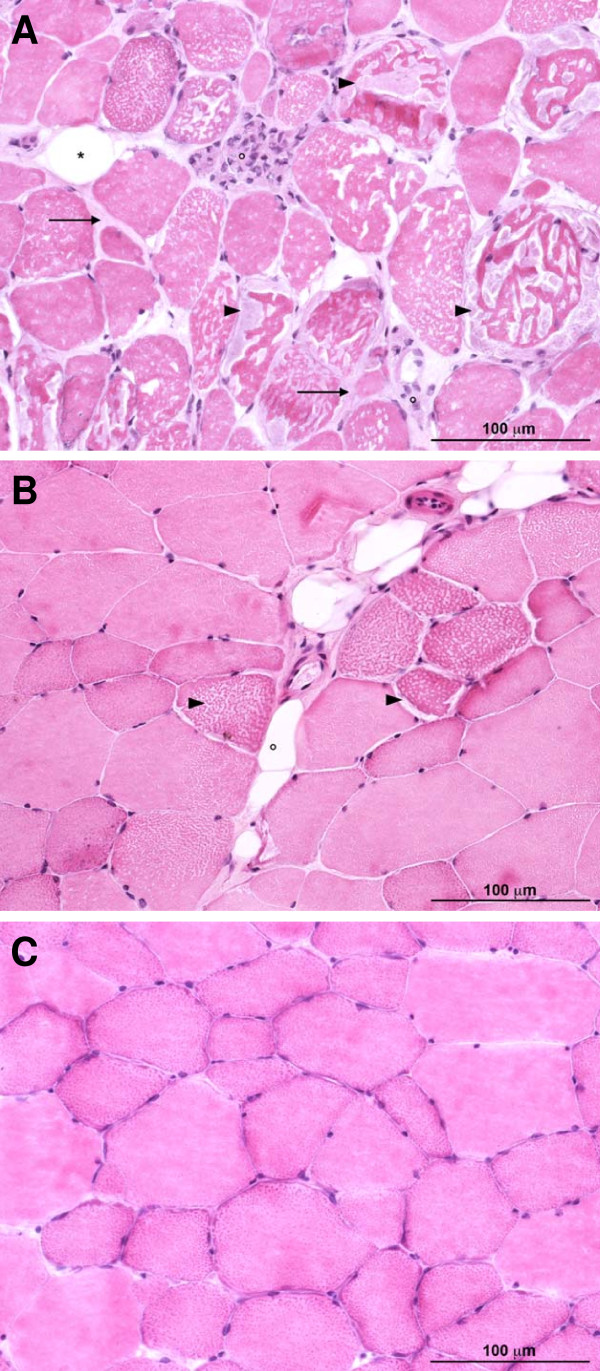
**Histological evaluation of muscular biopsies from Norman Cob horses**. (A) Severe form of polysaccharide storage myopathy corresponding to a homozygous horse (# 44) for the GYS1 c.926 A allele. Numerous muscle fibers contained abnormal accumulation of polysaccharidic material (arrowheads). The endomysium was focally infiltrated by mononuclear cells (°), some adipocytes (*) and was diffusely thickened by fibrosis (arrows). (B) Mild form of polysaccharide storage myopathy corresponding to a heterozygous horse (# 54) for the GYS1 c.926 A allele. Compared to the severe form of the disease, the number of polysaccharide containing fibers (arrowheads) was lower and the endomysium was subnormal except for some fatty infiltration (°). (C) Muscle aspect of a control horse (#56). (A-C) Hematoxylin-eosin-safranine staining.

Intracytoplasmic presence of amylase-resistant material, used as diagnosis criteria for PSSM, was observed in biopsies of the 7 out of the 13 horses (Figure 1). Two horses were graded as mildly affected (less than 3% of fibers containing PAS positive amylase resistant material) and five horses as severely affected (10 to 20% of affected fibers) (Table 2) using quantitative evaluation of aggregate-containing fibers as previously published [1]. The two homozygous horses were histologically severely affected. Heterozygous horses could present the mild (n = 2) or the severe (n = 3) phenotype.

Some other myopathic changes were also identified in affected horses with marked individual variations. Numerous fibers underwent segmental necrosis that was associated with mononuclear cell infiltration foci (Figure 1). Some of these foci were centered on fragmented fibers with many macrophages phagocytizing cellular necrotic debris. Strikingly, fiber necrosis was more prominent in homozygous PSSM horses. In this marked inflammatory context, some endomysial fibrosis was also present. Additionally, in all PSSM muscles, we noted an increased number of central nuclei. This change corresponds to muscular regeneration. Some degree of anisocytosis of muscular fibers was always present in affected muscles and was more severe in the two homozygous horses. Fatty infiltration of muscular fascia and replacement of muscle fibers by adipose tissue was also sometimes observed, this change being particularly severe in one homozygous horse.

**Figure 2:**
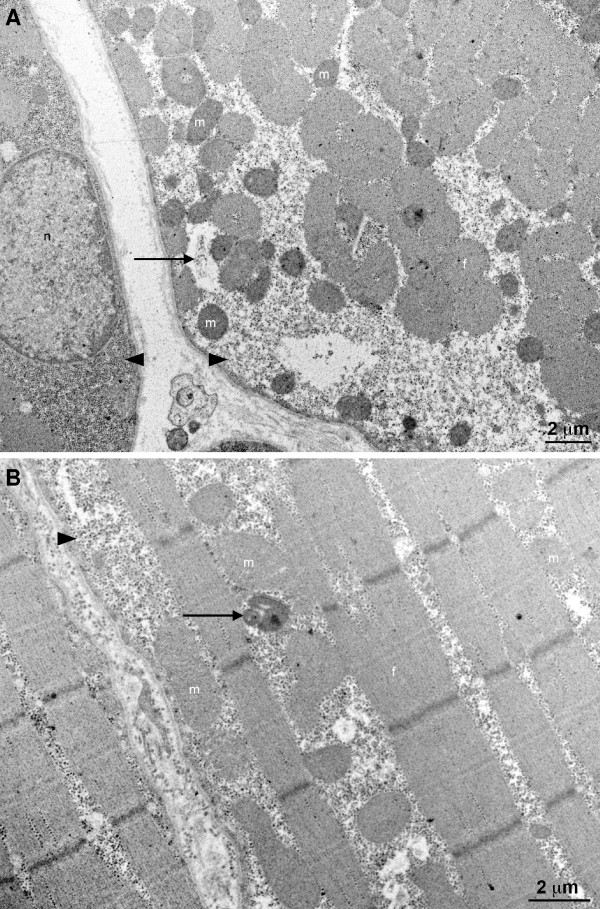
**Ultrastructural evaluation of muscular biopsy from a PSSM affected Norman Cob horse #49**. (A) Transversal section. (B) Longitudinal section. Severe mitochondrial (m) and myofibrillar (f) loss due to abnormal accumulation of granular material (arrowhead) resembling glycogen. Some myelinic bodies (arrow) were present that demonstrated mitochondrial degeneration. A nucleus is indicated (n).

Ultrastructural evaluation of one severely affected horse (#49) was performed (Figure 2). Extensive accumulation of an abnormal polysaccharide appearing as dense particles, displaced and partially replaced mitochondria and myofibrils, a number of which was severely decreased. This accumulation was exclusively cytoplasmic, membrane unbound and particularly abundant around nuclei and in the subsarcolemmal region of the cytoplasm, where numerous mitochondria are normally present in sound animals. Strikingly, we also reported some mitochondrial ultrastructural changes, namely a decrease of the cristae number, formation of myelinic bodies and swelling.

### Gene transcript expressions

Thirty-three percent of the probes of the micorarray were significantly hybridized with a high fluorescence value, especially for mitochondrial genes. All the data of the present study have been deposited in NCBI's Gene Expression Omnibus and are accessible through GEO series accession number GSE15572 .

After filtering the normalized expression ratios with a cut-off at p < 0.05 (normalized ratio significantly different from 1), a total of 129 genes exhibited significant changes of expression levels between PSSM and control muscles (Table 3). This set contained 16 up-regulated genes (normalized ratio > 1.5), 37 down-regulated genes (normalized ratio < 0.66) and 76 moderately modulated genes (0.66 < normalized ratio < 0.9 and 1.07 < normalized ratio < 1.5). This list of significant genes was used for the rest of the study. The maximal up-regulation value was observed for interleukin 18 (IL18) 6.2 fold change, and the following genes were up-regulated up to 2 fold: CTSS, LUM, CD44, FN1, GST01. The maximum down regulation was observed for the mitochondrial tRNA genes (TRNQ, TRNC, TRNN, TRNY, TRNA) from -4.83 to -2.86 fold changes. The three most down-regulated genes coding for proteins were the following: SLC2A2 or GLUT2 (-2.84), PRKCα (-2.72), VEGFα (-2.68).

**Table 3:** List of significant modulated genes (p < 0.05) expressed in the muscles suffering from PSSM.

**Gene name**	**EST – Sequence**	**Entrez Gene**	**Normalized ratio**	**P-value < 0.05**
IL18	Y11131	3606	6.20	1.58E-03

CTSS	CD464451	1520	3.17	3.24E-03

LUM	CX594607	4060	3.15	8.40E-04

CD44	CD464887	960	2.42	9.56E-04

FN1	ECU52108	2335	2.17	5.10E-03

GSTO1	CX603867	9446	2.03	2.64E-03

VCAM1	CX602484	7412	1.98	2.76E-02

ITGB1	CX598727	3688	1.87	2.31E-03

CXCR4	CD466324	7852	1.81	2.74E-02

HIF1A	CX602270	3091	1.73	1.36E-03

ITGB2	CD535228	3689	1.70	4.26E-04

HSPA5	CX604607	3309	1.69	1.42E-02

GNAS	BM734975	2778	1.63	1.45E-03

CD69	CD528606	969	1.62	2.67E-02

LTF	ECA010930	4057	1.58	1.73E-02

GSK3B	CX592808	2932	1.50	2.60E-03

APP	CX601350	351	1.40	1.04E-02

CAT	CD464332	847	1.39	6.36E-03

ENO1	CX603657	2023	1.39	7.60E-03

VAV1	BI961759	7409	1.37	2.70E-03

MYLK	CX592941	4638	1.35	5.21E-02

EPO	AB100030	2056	1.34	6.91E-03

IL15	AY682849	3600	1.34	4.57E-03

ITGAL	CD464254	3683	1.32	1.32E-02

CD40	AY514017	958	1.30	4.73E-02

ACTN2	AB178951	88	1.29	1.76E-02

GDF8	AB033541	2660	1.29	4.87E-02

DAG1	CX600578	1605	1.28	1.20E-02

IGF2	U11241	3481	1.28	2.61E-02

HCK	BM734694	3055	1.27	2.45E-02

ACAA2	CX603916	10449	1.25	1.67E-02

COMT	AB178284	1312	1.23	4.70E-04

FLII	CX604876	2314	1.21	2.73E-02

ATP5a1	DN509143	498	1.21	5.18E-02

MYL9	CX594102	10398	1.18	1.56E-02

CCL13	ECA251188	6357	1.17	1.04E-02

GJA5	AY008788	2702	1.07	5.07E-02

ATP5C1	CX593263	509	0.90	4.87E-02

PYGL	CX597788	5836	0.90	2.66E-02

FGR	CD470584	2268	0.88	4.71E-02

MRPL49	CD469126	740	0.87	5.00E-02

GOT2	CX602317	2806	0.87	4.82E-02

ALOX5AP	CD536322	241	0.86	1.95E-02

IMMT	CX597479	10889	0.85	4.56E-02

ECHS1	CX604357	1892	0.85	4.05E-03

SLC2A1	DQ139875	6513	0.85	6.96E-03

CYTB	X79547	4519	0.85	1.98E-02

EDN1	AY730629	1906	0.84	4.03E-02

PNMT	AB071422	5409	0.84	2.08E-03

IL7	CX592622	3574	0.84	4.32E-02

IDH2	CX604713	3418	0.83	1.09E-02

PTGER4	DN505656	5734	0.83	1.72E-05

IL6ST	DN507511	3572	0.83	4.34E-04

SCN4A	ECU25990	6329	0.82	3.11E-02

CEBPB	CX605423	1051	0.82	8.99E-03

SHMT2	CX605845	6472	0.82	2.08E-03

GLS	CX600244	2744	0.81	7.01E-03

CYP19A1	ECA012610	1588	0.81	3.23E-02

PLAU	CD471288	5328	0.81	3.89E-03

SLC25a5	DN506810	292	0.81	5.66E-03

ATP5o	DN509617	539	0.80	1.98E-02

NQO1	DN508254	1728	0.80	2.44E-04

FYN	CD528430	2534	0.78	1.17E-02

SLC8A1	DQ178640	6546	0.78	1.98E-03

HNRPK	CX598401	3190	0.77	4.69E-05

Leu-ARNt_TRNL1	X79547	4567	0.77	1.58E-03

MIF	CX599697	4282	0.77	1.05E-02

ND2	X79547	4536	0.76	3.34E-03

CYTB	X79547	4519	0.76	7.85E-03

OPRM1	ECA519535	4988	0.76	1.07E-04

CSF1	CD469550	1435	0.75	7.13E-04

RELB	CD472197	5971	0.75	5.86E-05

Met-ARNt_TRNM	X79547	4569	0.74	3.31E-03

SSPN	CX605718	8082	0.72	1.65E-02

COX 3	X79547	4514	0.72	5.08E-03

CPT2	CX599757	1376	0.72	1.49E-04

PDE4B	CD470720	5142	0.72	4.70E-06

ATP5e	DN509652	514	0.72	1.22E-02

NR3C1	CX597756	2908	0.72	1.89E-02

STAT6	CD467276	6778	0.72	1.91E-02

ND2	X79547	4536	0.72	1.09E-04

COX 3	X79547	4514	0.70	2.46E-03

ND6	X79547	4541	0.70	4.69E-05

OXTR	BM734870	5021	0.69	9.30E-04

ATP1B1	DN511201	481	0.69	2.20E-04

ATP5j	DN507306	522	0.69	4.03E-02

PLG	BM780461	5340	0.69	3.21E-04

COX 2	X79547	4513	0.69	1.03E-02

PKM2	CX604027	5315	0.69	1.82E-02

ATP5h	CX599509	10476	0.69	1.20E-03

ATP5d	CX603287	513	0.69	4.65E-04

CSF3	AF503365	1440	0.68	1.84E-05

Phe-ARNt_TRNF	X79547	4558	0.67	4.07E-05

Glu-ARNt_TRNE	X79547	4556	0.67	2.98E-06

TNFRSF4	CD465063	7293	0.66	6.88E-04

IGHE	ECA305046	3497	0.66	4.10E-05

ATP5l	CX603854	10632	0.66	4.69E-03

UCP2	CD528532	7351	0.66	8.40E-03

Lys-ARNt_TRNK	X79547	4566	0.65	1.60E-03

ND3	X79547	4537	0.64	1.12E-03

ND6	X79547	4541	0.63	7.60E-05

ATP2A1	AF489278	487	0.62	5.98E-04

TNFSF13B	CD470712	10673	0.62	1.95E-05

Gly-ARNt_TRNG	X79547	4563	0.62	4.97E-03

NFKBIB	CX601294	4793	0.59	9.31E-06

PLCG2	CX603458	5336	0.58	7.89E-08

ND5	X79547	4540	0.57	1.01E-05

ND3	X79547	4537	0.57	1.65E-05

SLC25a17	CX598253	10478	0.57	2.13E-06

SRI	DN506802	6717	0.57	4.60E-06

Val-ARNt_TRNV	X79547	4577	0.56	1.95E-04

Asp-ARNt_TRND	X79547	4555	0.55	1.76E-05

ND5	X79547	4540	0.54	1.39E-05

PDE4D	CX593241	5144	0.54	1.26E-06

Ser-ARNt_TRNS2	X79547	4575	0.50	6.05E-03

Ile-ARNt_TRNI	X79547	4565	0.49	1.21E-03

SLC25a15	CX598538	10166	0.48	4.95E-02

Leu-ARNt_TRNL2	X79547	4568	0.44	1.13E-05

Trp-ARNt_TRNW	X79547	4578	0.40	2.13E-06

Thr-ARNt_TRNT	X79547	4576	0.40	5.63E-05

Pro-ARNt_TRNP	X79547	4571	0.39	2.59E-06

VEGF	AB053350	7422	0.37	1.32E-06

PRKCA	CX602892	5578	0.37	2.18E-06

SLC2A2	AJ715983	6514	0.35	2.18E-06

Ala-ARNt_TRNA	X79547	4553	0.35	1.10E-04

Tyr-ARNt_TRNY	X79547	4579	0.35	1.04E-03

Asn-ARNt_TRNN	X79547	4570	0.33	8.13E-06

Cys-ARNt_TRNC	X79547	4511	0.28	4.48E-06

Gln-ARNt_TRNQ	X79547	4572	0.21	3.81E-07

Significant p-value (p < 0.05) indicated that the expression ratio was significantly different from 1.

P-values are expressed in scientific format: 1E-3 means 1.10^-3^.

The relative expression ratio of the top ten genes up- or down-regulated between PSSM and the control group were compared to the results obtained by microarray analysis (Table 4). A significant correlation of r = 0.54 (p < 0.01) between the two methods was calculated. As in microarray results, the mitochondrial genes ND3 and ND5 were found down-regulated using quantitative RT-PCR analysis. The hypoxia markers were also in agreement: VEGFα was down-regulated and HIF1α was up-regulated. All inflammation markers (IL18, CTSS, LUM, CD44, GSTO1, VCAM1, ITGB1, CXCR4) were up-regulated as in microarray results except FN1 and ITGB1 which were found down-regulated. The gene markers of aerobic pathway SLC25a17 was found up-regulated by quantitative RT-PCR and SCL25a15 down-regulated as in microarray data.

**Table 4:** Real time quantitative RT-PCR Results of the top ten up- and down-regulated genes measured by the microarray analysis.

**Gene**	**Normalized ratio of microarray analysis**	**Relative expression ratio of quantitative RT-PCR**	**RT-PCR ratio different from 1 p-value**
**UP**			

IL18	6.20	4.29	0.01

CTSS	3.17	1.61	0.05

LUM	3.15	1.75	0.05

CD44	2.42	2.87	0.01

FN1	2.17	0.72	0.05

GSTO1	2.03	3.23	0.01

VCAM1	1.98	7.21	0.01

ITGB1	1.87	0.09	0.05

CXCR4	1.81	3.43	0.01

HIF1A	1.73	2.99	0.01

**DOWN**			

NFKBIB	0.59	0.60	0.05

PLCG2	0.58	0.97	NS

ND3	0.57	0.60	0.05

SLC25a17	0.57	1.77	0.05

SRI	0.57	0.98	NS

ND5	0.54	0.49	0.05

PDE4D	0.54	0.67	0.05

SLC25a15	0.48	0.96	NS

VEGF	0.37	0.35	0.05

PRKCA	0.37	0.16	0.05

SLC2A2	0.35	0.79	0.05

One sample T-test compared the relative expression ratio of RT-PCR results against 1. For p > 0.05,

P-values were non-significant (NS).

### Gene functions and pathways

**Figure 3:**
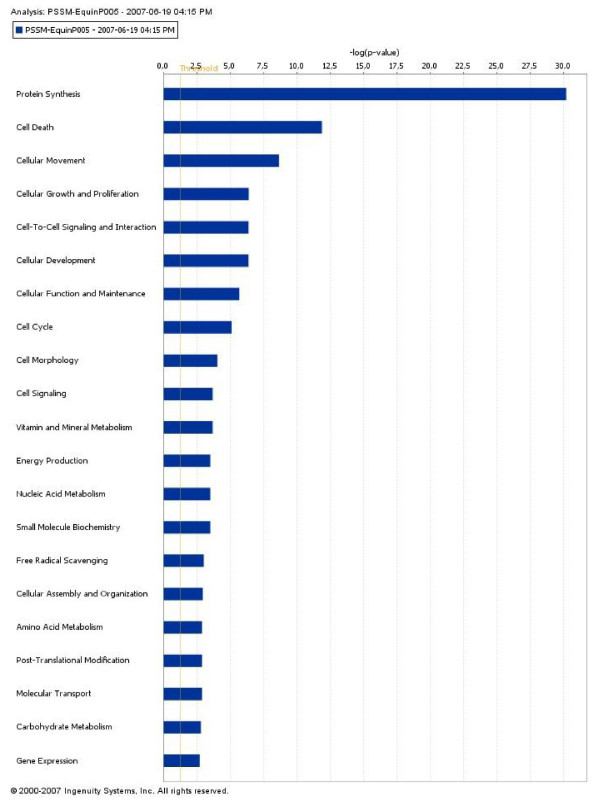
**Categories of cellular gene functions**. Our list of modulated genes was classified by categories of cellular functions using a data mining software (Ingenuity Pathway Analysis^®^). The analysis identified the biological functions that were the most significant to the gene list results. The significant genes associated with biological functions in the knowledge database (gene functions described in published papers) were considered for the analysis. The Fischer exact test was used to calculate a p-value determining the probability that each biological function assigned to that data set is due to chance alone. In the present figure, protein synthesis, apoptosis, cellular movement, growth and proliferation are the main significant cellular functions (p < 0.05) that are associated to the modulated genes.

Our list of modulated genes was classified by categories of cellular functions, canonical pathways and signaling pathways using Ingenuity Pathway Analysis^®^. Protein synthesis, apoptosis, cellular movement, growth and proliferation are the main significant cellular functions (p < 0.05) that are associated to the modulated genes (Figure 3). The main significant (p < 0.05) canonical pathways concerned oxidative phosphorylation, ubiquinone biosythesis, aminosugar and purine metabolism. The main significant signaling pathways (p < 0.05) were related to G-protein couple receptor, hypoxia, NF-κB, VEGFα and IL6, IL10, apoptosis and leukocyte extravasation signaling. The main metabolic disorders significantly (p < 3.3 10^-4^) detected were mitochondrial dysfunction, hypoxia and alteration of NF-κB activity.

**Figure 4:**
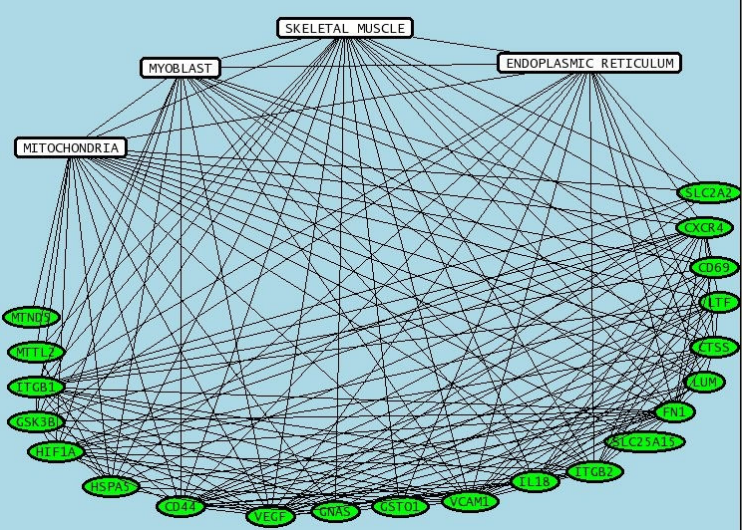
**Relationships between pairs of genes and cellular compartments**. The analysis of the main metabolic pathways and their dysfunctions was performed using a data mining software (Predictsearch^®^). Briefly, gene aliases corresponding to the significant modulated genes were submitted as queries to PubMed in order to collect titles and abstracts of all related publications. The results showed that a part of the significant modulated genes were strongly associated with the following terms: skeletal muscle, mitochondria, myoblast, endoplasmic reticulum.

**Figure 5:**
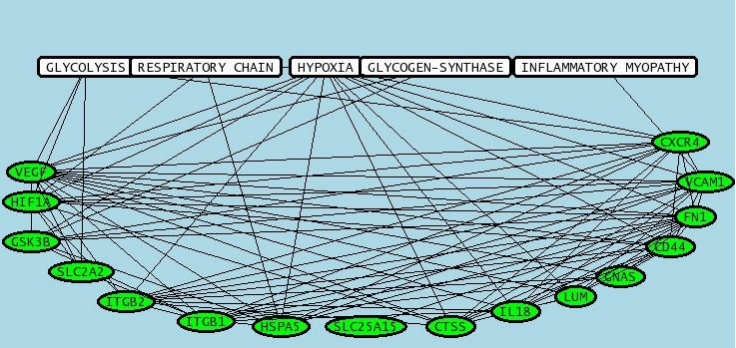
**Relationships between metabolic pathways or disorders and genes**. The analysis of the main metabolic pathways and their dysfunctions was performed using a data mining software (Predictsearch^®^). Briefly, gene aliases corresponding to the significant modulated genes were submitted as queries to PubMed in order to collect titles and abstracts of all related publications. The PubMed ID of the related publications were indicated on the figures 6, 7 and 8. The results showed that the more frequent biological activities cited in relationship with the set of genes were glycolysis, respiratory chain, hypoxia, glycogen synthase and inflammatory myopathy.

**Figure 6:**
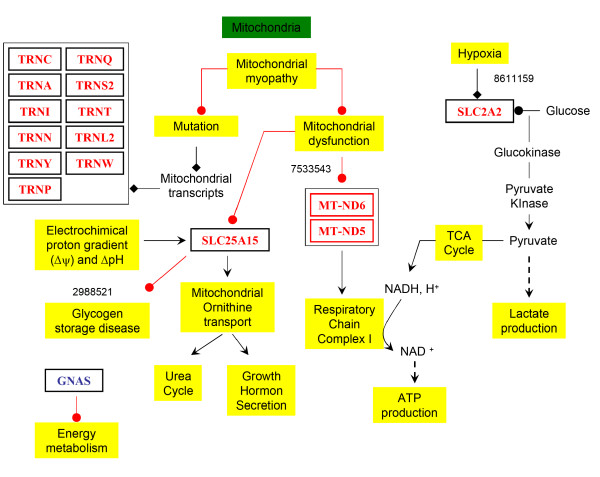
**Mitochondrial dysfunctions**. Mitochondria is one of the compartment detected by electronic microscopy and data mining as illustrated in figure 4. In the mitochondria, the tRNA were severely down regulated (fold change between -4.83 and -2.27). The other mitochondrial genes ND2, ND3, ND5, ND6, COX2 and COX3 were moderately down-regulated but with low expression values (fold changes between -1.31 and -1.85). Other nuclear genes involved in the aerobic metabolism of the mitochondria were down regulated as well: UCP2, GNAS, SLC25a15, SLC2a2 (GLUT2), ATP5L, ATP5J, ATP5D and ATP5H. The PubMed ID of the related publications found by the data mining analysis were indicated on the figure (8611159; 7533543; 2988521).

**Figure 7:**
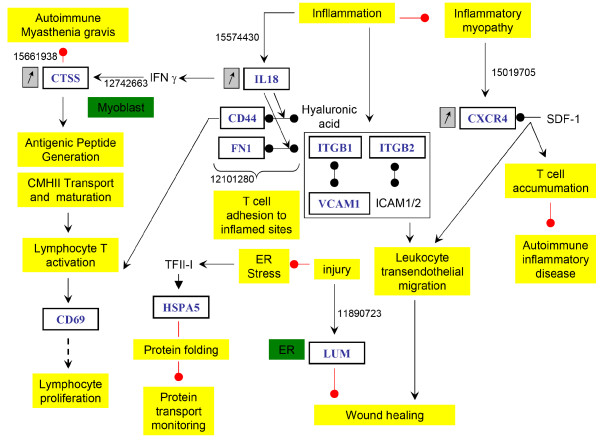
**Inflammatory process**. The chronic inflammation of the PSSM muscle was revealed both by histological observations and the number of up-regulated genes involved in inflammation pathways as illustrated in data mining results figure 5: IL18, CD44, CD69, CTSS, FN1, HSPA5, ITGB1, ITGB2, VCAM, CXCR4, LTF, GSTO1 and LUM. These genes could be expressed in the leukocytes present in the PSSM muscle and/or in muscle fibers. The PubMed ID of the related publications found by the data mining analysis were indicated on the figure (15574430; 15661938; 12742663; 12101280; 15019705; 2988521; 11890723).

**Figure 8:**
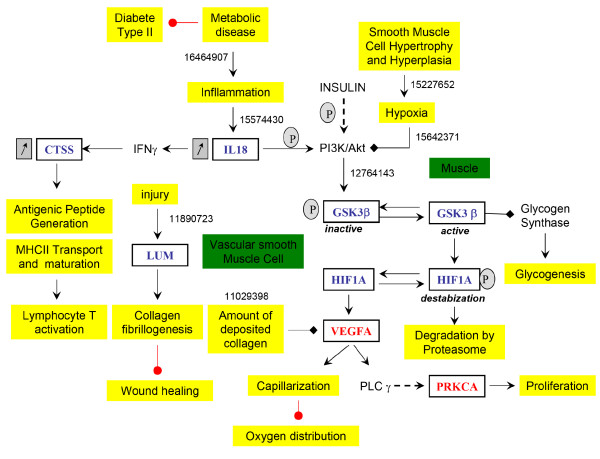
**Relationships between inflammatory process, inhibition of glycogen synthase and hypoxia according to data mining Results of figure 5**. The up-regulation of the glycogen synthase kinase (GSK3β) under its active form was responsible for the glycogen synthase (GYS1) inhibition. The up-regulation of GSK3β has another effect on hypoxia and low capillarization of the PSSM muscle. Phosphorylation of GSK3β increases the HIF1α destabilization which is degraded in the proteasome. Consequently, the low expression of HIF1α down-regulates VEGFα expression which contributes to a poor capillarization and increases chronic hypoxia of the regenerated muscle fibers. The PubMed ID of the related publications found by the data mining analysis were indicated on the figure (16464907; 15574430; 15227652; 15642371; 12764143; 11890723; 11029398).

The main tissue, organ and cellular context and the biological activities that were cited in relationship with the set of genes were further investigated using Predictsearch^® ^analysis. Using the PubMed database, it was found that a part of the significant modulated genes were strongly associated with the following terms: skeletal muscle, mitochondria, myoblast, endoplasmic reticulum. (Figure 4). In addition, the more frequent biological activities cited in relationship with the set of genes were glycolysis, respiratory chain, hypoxia, glycogen synthase, inflammatory myopathy (Figure 5). The main metabolic pathway disorders are described in the figures 6, 7 and 8 using these data (see Additional file 2 for the legends of the pathway drawings). In the mitochondria, all the genes were down regulated (Figure 6). The tRNA were severely down regulated (fold change between -4.83 and -2.27). The other mitochondrial genes ND2, ND3, ND5, ND6, COX2 and COX3 were moderately down regulated but with low expression values (fold changes between -1.31 and -1.85). Other nuclear genes involved in the aerobic metabolism of the mitochondria were down regulated as well: UCP2, GNAS, SLC25a15, SLC2a2 (GLUT2), ATP5L, ATP5J, ATP5D and ATP5H.

A chronic inflammation process of the muscle was revealed by many up-regulated genes like interleukin 18 (IL18), CD44, CD69, CTSS, FN1, HSPA5, ITGB1, ITGB2, VCAM, CXCR4 LTF, GSTO1 and LUM (Figure 7).

The up-regulation of the glycogen synthase kinase-3 (GSK3β) under its active form could be responsible for glycogen synthase inhibition, and for hypoxic transcription factor inhibition (HIF1α) by phosphorylation as indicated by data mining analysis (Figure 8). In addition, VEGFα was down-regulated and avoided capillarization and oxygen distribution of the new regenerated muscle fibers. These gene regulations increased muscle hypoxia.

Other interesting genes were slightly up-regulated: ENO1 (1.39) was involved in the end of glycolysis; IGF2 (1.34) was involved in the growth hormone activity in muscle differentiation; EPO (1.28) was involved in hematopoietic function. Some other genes were slightly down-regulated: PYGL (-1.11) was involved in glycogen metabolism; IL6ST (-1.20), IL7 (-1.19) were involved in hematoprotein signaling; MIF (-1.30) was involved in cell inflammatory regulation. GSK3β, PYGL, GLUT2 are the only genes closely related to glycogen metabolism.

## Discussion

The equine genome has been recently sequenced and published. However, there is no equine DNA microarray commercially available to date. Three homemade cDNA microarrays have been designed using transcripts extracted from equine joint cartilage and synoviocytes [27-29]. This first cDNA microarray was tissue specific and poorly annotated. Another approach to analyse gene expression in equine leukocytes and muscles was to use a heterologous 15 K cDNA microarray designed from murine embryonic tissues by the National Institute on Aging [30]. It includes 15,264 unique genes for which about 11,000 are annotated. It has been demonstrated by several tests that mouse cDNA microarrays can adequately hybridize equine genes expressed in muscles [31]. It was assumed that interspecific hybridization between an equine cDNA and a probe of 1,500 nucleotide length is possible between two mammal species because the genes involved in the basic cellular functions are highly conserved during mammal evolution as observed for myosin heavy chains [32]. The same mouse 15 K cDNA microarray has been used to detect inflammation and oxidative stress by the study of leukocyte gene expression in endurance horses performing a race of 140 km [33]. In order to improve specificity of the hybridization and to focus on a few numbers of functions of the genes (inflammation, muscle metabolism), an equine oligonucleotide microarray database was designed and validated according to the same methods as previously described [24,31].

The muscle biopsies mainly collected muscle fiber cells and also some leucocytes that were in the muscular blood vessels and in the muscle connective tissue itself because of chronic inflammation observed in PSSM muscles. Histological data showed mild or severe inflammation with macrophages and lymphocyte infiltration. These leucocytes should have an important signaling activity which was observed in the present results. However, the relative amount of muscle RNA extracted from the muscle was more than 98% of the total RNA according to the relative mass of tissue. In addition, very little blood was collected with the automatic biopsy needle and the blood at the surface of the sample was absorbed before freezing the biopsy into nitrogen.

The gene expression analysis of the PSSM muscles mainly revealed the metabolic disorders due to this equine glycogenosis but not directly the genes suspected to be responsible for this pathology. The candidates GBE1, PFKM, AGL and even GYS1 were not significantly modulated. GYS1 with a G-to-A mutation has been identified to be associated with PSSM in different breeds [21]. Most of the PSSM cases (77) diagnosed by histology were heterozygous for the mutation but 22 PSSM cases were of the homozygous wild type and the GYS1 mutation alone could not explain the glycogenosis. It has been suggested that another non-GYS1 glycogenosis could explain these 22 PSSM cases. The glycogen synthase activity measured in vitro was higher in PSSM muscle. In the present study, all the PSSM horses were mutated for GYS1. The homozygous horses exhibited more severe histological disorders with characteristic amylase-resistant aggregates but also tissular damages, namely fiber necrosis, anisocytosis, some endomysial fibrosis and fatty infiltration.

The main dysfunctions observed were a chronic inflammatory myopathy and a severe hypoxia associated with a very low activity of the oxidative energetic metabolism. The chronic inflammation of the PSSM muscle was revealed both by histological observations and the number of up-regulated genes involved in inflammation pathways: IL18, CD44, CD69, CTSS, FN1, HSPA5, ITGB1, ITGB2, VCAM, CXCR4, LTF, GSTO1 and LUM. These genes could be expressed in the leukocytes present in the PSSM muscle and/or in muscle fibers. Interleukin 18 (IL18) was the most up-regulated gene and four other genes had the highest expression ratios: CTSS, LUM, CD44 and FN1. The chemokine receptor 4 (CXCR4) has been observed more specifically in some idiopathic inflammatory myopathies in humans [34]. This gene encodes a CXC chemokine receptor specific for stromal cell-derived factor-1. This protein has 7 transmembrane regions and is located on the cell surface.

According to the histological data, it was postulated that this inflammatory context was stimulated by muscle fiber necrosis and progressed to fiber regeneration and endomysial fibrosis. This mecanism is illustrated by (i) the insulin like growth hormone type II (IGF2) that was also up-regulated and contributed to myoblast differentiation [35] and mitochondrial biogenesis [36]; (ii) the up-regulation of lumican (LUM) gene that encodes for a small keratan sulfate proteoglycan molecule, which is involved in the collagen fibrillogenesis and fibrous tissue formation.

IL18 is an interferon gamma-induced protein that is involved in immune cell activation, proliferation, infiltration, apoptosis and cytotoxicity. In humans, the plasmatic concentration of IL18 is an interesting predictor of the risk of myocardial infarction, artherosclerosis and metabolic syndrome observed in obesity [37,38]. A high plasmatic concentration of IL18 is correlated with insulin resistance and body mass index. In PSSM horses, an increased sensitivity to insulin has also been observed [14]. The measure of IL18 concentration could be an interesting blood marker of PSSM. As indicated by the data mining analysis, the chronic inflammation and IL18 up-regulation could be responsible of PI3K/Akt/GSK3β activation [39]. The up-regulation GSK3β under its active form could be responsible for the glycogen synthase (GYS1) inhibition as indicated by data mining analysis. The down-regulation of the glycogen metabolism was also indicated by a low expression value of PYGL. The up-regulation of active GSK3β could be responsible of hypoxia and low capillarization of the PSSM muscle. The GSK3β activity increases the HIF1α destabilization by phosphorylation which is finally degraded in the proteasome [39,40]. Consequently, the low expression of HIF1α should down-regulate VEGFα expression [41] which contributes to a poor capillarization and increased chronic hypoxia of the regenerated muscle fibers. The down-regulated protein kinase Cα (PRKCα) gene is involved in many important signaling pathways like calcium, MAPK and VEGFα.

Other genes involved in hematoprotein signaling were down-regulated IL6ST, IL7. It was interesting to observe the up-regulation of the erythropoietin cytokine gene (EPO), which also demonstrated hypoxia in the PSSM muscle. This EPO transcript could be expressed both in blood and muscle cells. This plasmatic cytokine is well known to regulate red cell production by promoting erythroid differentiation and initiating hemoglobin synthesis. This cytokine also has neuroprotective activity and an anti-apoptotic neuronal effect useful for treatment of a variety of potential brain injuries [42,43]. The use of EPO in chronic kidney disease [44], heart failure and other cardiovascular diseases [45] demonstrated the pleiotropic cytokine activity of EPO as anti-apoptotic and with a tissue protective effect against hypoxia. Erythropoietin injection could be useful for the treatment of hypoxia in PSSM cases.

Hypoxia could also be related to the severe oxidative metabolism dysfunction observed at the mitochondrial level. Ultrastructural observations showed a decrease in the number of mitochondria and mitochondrial damages with reduction the cristae number, swelling and formation of myelinic bodies. The genomic markers of the mitochondrial dysfunction were the low level of most of the tRNA and the down-regulated genes coding for the respiratory chain sub-units (ND2, ND3, ND5, ND6, COX2 and COX3). The respiratory chain dysfunction in conjunction with a low expression of UCP2 could increase the reactive oxygen species accumulation in the mitochondria and could be responsible for muscle fiber apoptosis and necrosis. In addition, other nuclear genes involved in the mitochondrial oxidative metabolism were down-regulated (GNAS, SLC25A15, SLC2a2 (GLUT2), ATP5L, ATP5J, ATP5D and ATP5H). The down-regulation of the glucose transporter GLUT2 could also be related to the hypoxia. This transporter facilitates bidirectional transport of glucose through the cellular membrane and decreases the glucose available for glycogen synthesis, glycolysis and the Krebs cycle. The mitochondrial carrier ornithine transporter (SLC25A15) down-regulation also indicates a reduction of mitochondrial activity.

## Conclusion

Protein synthesis, apoptosis, cellular movement, growth and proliferation were the main cellular functions associated with the modulated genes. This was in agreement with the histological observations of active muscle fiber regeneration in PSSM muscles. Several up-regulated genes, especially interleukine 18 (IL18), revealed a severe muscular inflammation in PSSM muscles as confirmed by histological findings of leucocyte infiltrations. The up-regulation of active glycogen synthase kinase-3 (GSK3β) could be responsible for both glycogen synthase (GYS1) inhibition and hypoxia-inducible factor (HIF1α) destabilization. Many genes involved in mitochondrial functions were down-regulated and utltrastructural observations revealed many mitochondrial disorders. Consequently, the main disorders observed in PSSM muscles could be related to mitochondrial dysfunctions, glycogenesis inhibition and the chronic hypoxia.

## Authors' contributions

EB designed and carried out the study and wrote the manuscript. EM carried out the RNA extraction and microarray hybridization. NJ carried out the equine microarray design. TL carried out the histopathological and ultrastructural analysis. LG carried out the histological processing. BH and SC collected the PSSM cases and muscle biopsies. GG participated in the collection of PSSM cases and data analysis. XM participated in the quantitative RT-PCR analysis. PB and MC participated in the data mining analysis. OA and PM produced the home made equine microarrays and check quality. XG participated in the equine microarray design and the functional genomic analysis of the data. All authors read and approved the final manuscript.

## Supplementary Material

Additional file 1 **List of the 384 equine gene probes included in the equine microarray**. The file provides the following information for each probe: the gene name, Genebank ID, Entrez Gene ID of the human orthologous gene and location of the 50nt-oligonucleotides in the equine transcript sequence. More information is available on GEO database under accession number GPL8349: . Click here for file

Additional file 2 **Legend of metabolic pathways presented in figures 6, 7 and 8. **Signs and conventions used to describe the pathways presented in figures 6, 7 and 8. Click here for file
