# Supplementary material for: Gene expression profiling in equine polysaccharide storage myopathy revealed inflammation, glycogenesis inhibition, hypoxia and mitochondrial dysfunctions
Source: BMC Vet Res. 2009 Aug 7;5:29. doi: 10.1186/1746-6148-5-29 (PMC2741442; doi:10.1186/1746-6148-5-29)

Legends of figures

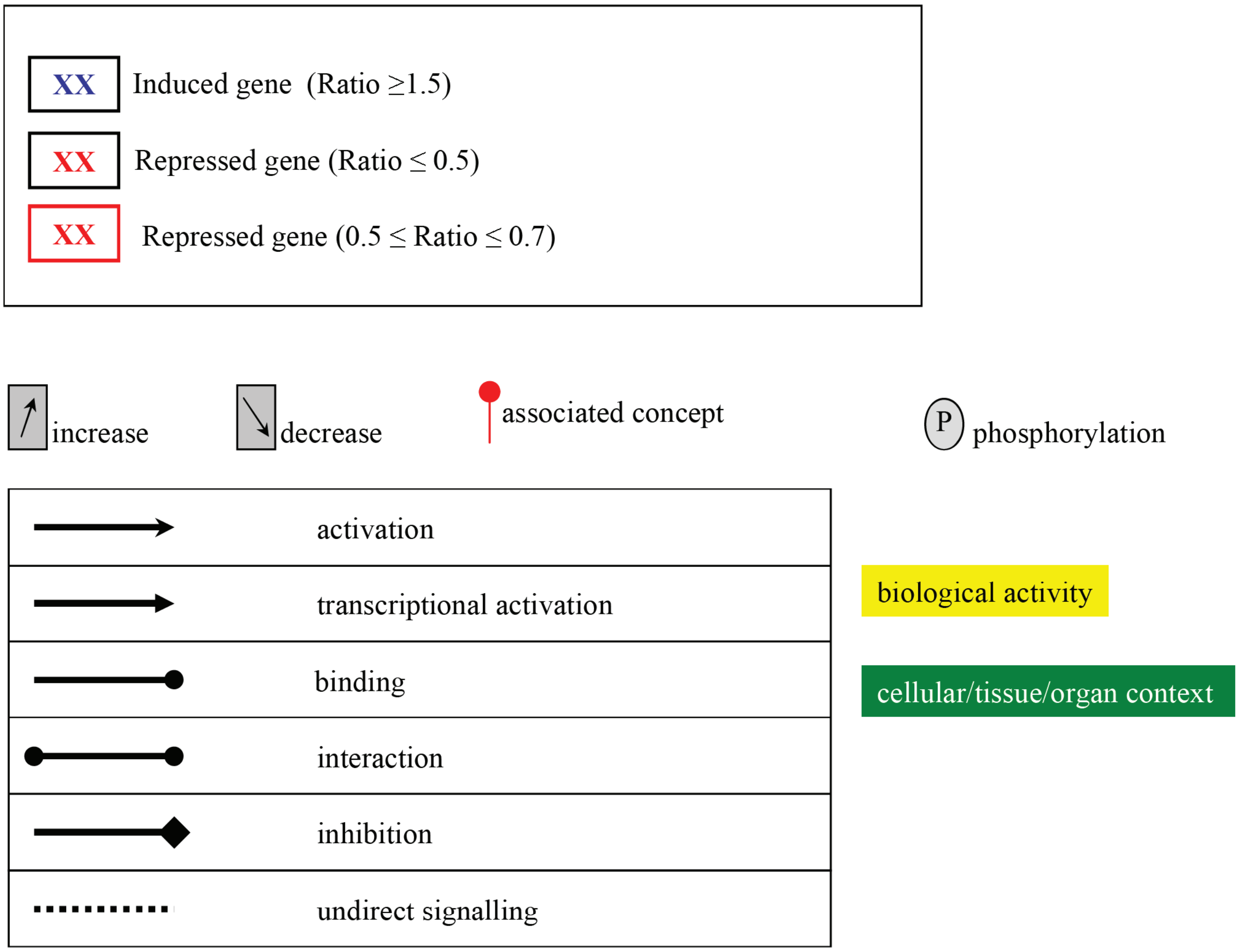

Supplement: Additional file 2 — Legend of metabolic pathways presented in figures 6, 7 and 8. Signs and conventions used to describe the pathways presented in figures 6, 7 and 8. [file 1746-6148-5-29-S2.pdf]
